# Synchrotron macro ATR-FTIR microspectroscopic analysis of silica nanoparticle-embedded polyester coated steel surfaces subjected to prolonged UV and humidity exposure

**DOI:** 10.1371/journal.pone.0188345

**Published:** 2017-12-18

**Authors:** Jitraporn Vongsvivut, Vi Khanh Truong, Mohammad Al Kobaisi, Shane Maclaughlin, Mark J. Tobin, Russell J. Crawford, Elena P. Ivanova

**Affiliations:** 1 Infrared Microspectroscopy (IRM) Beamline, Australian Synchrotron, Clayton, Victoria, Australia; 2 School of Science, Faculty of Science, Engineering and Technology, Swinburne University of Technology, Hawthorn, Victoria, Australia; 3 ARC Research Hub for Australian Steel Manufacturing, Wollongong, New South Wales, Australia; 4 BlueScope Steel Research, Port Kembla, New South Wales, Australia; 5 School of Science, College of Science, Engineering and Health, RMIT University, Melbourne, Victoria, Australia; VIT University, INDIA

## Abstract

Surface modification of polymers and paints is a popular and effective way to enhance the properties of these materials. This can be achieved by introducing a thin coating that preserves the bulk properties of the material, while protecting it from environmental exposure. Suitable materials for such coating technologies are inorganic oxides, such as alumina, titania and silica; however, the fate of these materials during long-term environmental exposure is an open question. In this study, polymer coatings that had been enhanced with the addition of silica nanoparticles (SiO_2_NPs) and subsequently subjected to environmental exposure, were characterized both before and after the exposure to determine any structural changes resulting from the exposure. High-resolution synchrotron macro ATR-FTIR microspectroscopy and surface topographic techniques, including optical profilometry and atomic force microscopy (AFM), were used to determine the long-term effect of the environment on these dual protection layers after 3 years of exposure to tropical and sub-tropical climates in Singapore and Queensland (Australia). Principal component analysis (PCA) based on the synchrotron macro ATR-FTIR spectral data revealed that, for the 9% (w/w) SiO_2_NP/polymer coating, a clear discrimination was observed between the control group (no environmental exposure) and those samples subjected to three years of environmental exposure in both Singapore and Queensland. The PCA loading plots indicated that, over the three year exposure period, a major change occurred in the triazine ring vibration in the melamine resins. This can be attributed to the triazine ring being very sensitive to hydrolysis under the high humidity conditions in tropical/sub-tropical environments. This work provides the first direct molecular evidence, acquired using a high-resolution mapping technique, of the climate-induced chemical evolution of a polyester coating. The observed changes in the surface topography of the coating are consistent with the changes in chemical composition.

## Introduction

The surface modification of polymers and paints used to coat substrate surfaces is an attractive method for enhancing the properties of these materials [1–4]. A thin overcoat can preserve the desirable properties associated with the bulk material, such as mechanical strength and appearance, but can change the way in which the surface interacts with the environment. One example is the application of titania films to substrates for the purposes of imparting photocatalytic, self-cleaning functionality to the surface [2]. Materials typically used for these applications are inorganic oxides, such as alumina, titania and silica [2–4]. These materials are often combined or derivatized in various ways; one example is via the absorption of functional agents throughout mesoporous networks. These coatings may be applied through solution based processes or via vapor deposition technologies. Typically, the thickness of the layers applied is less than one micron. Given that the substrate surfaces being coated may be unstable in the environment, the fate of the coating materials and their ability to retain their protective and mechanical properties during long-term environmental exposure is of paramount importance. Recent advances in surface engineering have seen the use of silica nanoparticles (SiO_2_NPs) in the development of innovative, highly functional and durable surface coatings that exhibit enhanced abilities to resist corrosion [5]. The long-term effect of environmental exposure upon these dual protection layers on steel surfaces has, however, not been studied.

The highly collimated synchrotron infrared (IR) beam essentially provides 100–1000 times greater brightness than those obtained using conventional thermal Globar™ IR sources in most laboratory-based FTIR instruments. This highly intense beam enables the acquisition of high-quality FTIR spectra at diffraction-limited spatial resolutions [6,7]. As such, the synchrotron FTIR technique represents an excellent analytical platform for acquiring spatially-resolved chemical mapping of materials at a lateral resolution between 3–10 μm (depending on the wavelength used). At the Australian Synchrotron IR beamline, a technique based on macroscopic (macro) attenuated total reflection (ATR)-FTIR microspectroscopy was recently developed to enable the coupling of the synchrotron IR beam to a germanium (Ge) ATR element [8]. The high refractive index of Ge (*n*_Ge_ = 4) allows an ATR-FTIR mapping measurement to be used to probe surface-specific molecular information pertaining to materials at four times greater resolution than that able to be achieved in comparable transmission and reflectance modes [8]. Unlike the traditional microscopic (micro) ATR-FTIR approach, the macro ATR-FTIR technique only requires a single contact to be made between the sample and the ATR crystal for the entire mapping measurement, which not only results in a greater scanning speed, but also reduces the possibility of sample damage and cross-contamination between measurement positions.

Our preliminary observation using the synchrotron ATR-FTIR microspectroscopic technique, performed at low resolution in the early stage of the development, indicated a decrease in the carbonyl (C = O) band intensity in the pure polyester coating with no SiO_2_NPs present after three years of exposure to the sub-tropical climate in Queensland (Australia) [9]. In this study, the chemical evolution occurring in SiO_2_NPs-embedded polyester composite coatings on steel substrata as a result of environmental factors was observed over time and analyzed at the molecular level using the synchrotron macro ATR-FTIR technique previously described. High-resolution chemical maps were acquired for the surfaces of polyester composite coatings containing different amounts of SiO_2_NPs. These samples were exposed to tropical and sub-tropical environments for a period of 3 years. A chemometric approach, specifically principal component analysis (PCA), was subsequently applied to the synchrotron macro ATR-FTIR spectral data, in order to determine specific changes in the molecular structure of the surface. These results were then compared to those samples that had not been environmentally exposed. The changes in the molecular structure, along with the corresponding surface topographic data obtained from optical profilometry and atomic force microscopy (AFM), provided an insight into changes taking place in the surface properties of the coating materials resulting from the environmental exposure. This information is critical in developing methods by which the durability of the polymer composite coatings can be improved. It should be noted that this work represents the first time that direct molecular evidence, performed using high-resolution synchrotron FTIR mapping technology, has been reported to highlight the chemical evolution of polyester composite coatings on steel surfaces as a result of environmental exposure.

## Materials and methods

### Materials

Steel disc samples with a pure polyester coating (0% SiO_2_NPs) and SiO_2_NPs-embedded polyester coatings (4.5% and 9% SiO_2_NPs) were supplied by Bluescope Steel Australia (New South Wales, Australia). The samples were subsequently exposed to both tropical (Singapore) and sub-tropical (Rockhampton, Australia) for 3 years. The relevant climate data were obtained from Meteorological Service Singapore and Bureau of Meteorology, Australian Government, respectively. The climate data is shown in S1 Fig. Prior to analysis, the steel disc samples were rinsed with double-distilled water and dried using nitrogen gas (99.99%). After drying, samples were kept in airtight containers to prevent any contamination of the surface.

### Atomic force microscopy

Atomic force microscopy (AFM) was performed using an Innova^®^ atomic force microscope (Bruker Corporation) in tapping mode using a phosphorus doped silicon probe with a spring constant of 0.9 N/m, tip curvature with a radius of 8 nm and a resonance frequency of 20 kHz [10]. As was the case with the optical profilometry studies, measurements were performed in triplicate on 3 individual coated steel discs per coating condition and on 3–4 different locations on each disc. The statistical parameters relating to surface roughness were then quantitatively determined over the nano-scale range using the standard instrument software. The results were expressed in terms of mean roughness together with the corresponding standard deviation, following commonly used protocols. Statistical data processing was performed using a paired Student’s two-tailed *t*-tests in order to evaluate the consistency of the results.

### Surface wettability measurement

Surface wettability measurements were conducted using a contact angle goniometer (FTA1000 C Class, First Ten Ångstroms Inc., VA, USA) equipped with a nanodispenser. The measurement was performed in triplicate on 3 individual coated steel discs for each coating condition and at 9–10 locations on each disc. Water contact angles were analyzed using the FTA1000C version 2.0 software [9,11]. An average value of the contact angle was obtained from at least five measurements, and expressed as a mean ± standard deviation.

### Synchrotron macro ATR-FTIR mapping microspectroscopy

Synchrotron macro ATR-FTIR microspectroscopy measurements were performed at Infrared Microspectroscopy (IRM) beamline (Australian Synchrotron), using a Bruker Vertex V80 v spectrometer coupled with a Hyperion 2000 FTIR microscope and a liquid nitrogen-cooled narrow-band mercury cadmium telluride (MCT) detector. An in-house developed macro ATR-FTIR device, equipped with a 250-μm-diameter facet Ge ATR crystal, was used to acquire the spectral maps of the polymer composite coating surfaces in the mapping mode.

In practice, the Ge ATR crystal was initially brought to the focus of the synchrotron IR beam, and a background spectrum was recorded in air using a spectral resolution of 4 cm^-1^ and 256 co-added scans. The coated steel disc was then mounted on an aluminum disc using double-sided polyimide (Kapton^®^) tape. This was then brought into contact with the flat sensing facet of the Ge ATR crystal. In order to locate areas on the surface that provided good contact, a low-resolution overview spectral map was initially measured to cover a 150 × 100 μm^2^ area at a 5 μm step interval using 8 co-added scans. A high-resolution map was subsequently acquired on the position where both strong FTIR signals were obtained according to the result from the prior low-resolution chemical map, and where interesting features were observed on the visible image. In all cases, the acquisition of a high-resolution map was obtained that covered a total area of 30 × 30 μm^2^ at a 1 μm step interval, using 16 co-added scans.

In this study, every spectrum was collected with a beam defining aperture that provided a nominal measurement area of 3.1 μm in diameter per pixel. For each pixel, the synchrotron macro ATR-FTIR spectrum was recorded within a spectral range of 3800–700 cm^-1^ using a spectral resolution of 4 cm^-1^. Default acquisition parameters were used, including Blackman-Harris 3-Term apodization, Power-Spectrum phase correction, and zero-filling factor of 2. These parameters were set using the OPUS 7.2 software suite (Bruker).

### Spectral pre-processing and multivariate data analysis

Atmospheric compensation was first performed on every synchrotron macro ATR-FTIR spectral map, using the OPUS 7.2 software suite (Bruker), to remove the presence of atmospheric water vapour and carbon dioxide from the spectra. The spectra embedded in each corrected spectral map were then pre-processed using CytoSpec v. 1.4.02 (Cytospec Inc., Boston, MA, USA) software, using noise reduction and 2^nd^ derivatization with 13-point Savitzky-Golay algorithms [12], in order to eliminate the broad baseline offset and curvature. Application of the 2^nd^ derivatization algorithm improves the performance of the subsequent multivariate data analysis by removing any non-chemical aspects, such as the optical and scattering artifacts that arise from the input spectra [13,14]. The 2^nd^ derivative spectra were then vector-normalized using only the spectral range that contain major bands relevant to the polyester coating components (i.e. 1790–970 cm^-1^), in order to remove the scaling effects that arise as a result of differences in sample thickness.

Finally, a multivariate pattern recognition method, based on a hierarchical cluster analysis (HCA) [15], was performed to discriminate the pre-processed 2^nd^ derivative spectra for each spectral map into five clusters using the same spectral range that contain the major bands associated to the polyester coatings (i.e. 1790–970 cm^-1^). It should be emphasized that in this study, HCA was used for screening and extracting good quality spectra from each spectral map, in order to ensure that the molecular characteristics of each coating group were appropriately represented. Accordingly, spectra with strong FTIR signals and high signal-to-noise (S/N) ratios were extracted from the selected HCA cluster(s) to be used for the subsequent PCA.

A principal component analysis (PCA) [16] was performed using The Unscrambler^®^ 10.1 software package (CAMO Software AS., Oslo, Norway). To measure the changes in molecular structure that resulted from subjecting the samples to environmental exposure, representative absorbance spectra were extracted from the selected HCA clusters of the coating groups with the same content of SiO_2_NPs before and after 3 years of exposure. These spectra were then combined and converted to 2^nd^ derivatives using a 13-point Savitzky-Golay algorithm. The total number of spectra that were extracted from each coating group, varied within 500–900 spectra. The resultant 2^nd^ derivative spectra were then corrected using an extended multiplicative scatter correction (EMSC) method [17] over the same spectral range (1790–970 cm^-1^). In principle, the EMSC pre-treatment is used in order to remove any light-scattering artefacts and to normalize the spectra, accounting for path-length differences. As a result, the EMSC-corrected 2^nd^ derivative spectra corresponded, in a more linear fashion, to the analyte concentration compared to that obtained using untreated spectra, leading to greater interpretability and an improved discriminatory accuracy of the multivariate data analysis approaches [14,17,18]. The PCA was performed using the combined EMSC-corrected 2^nd^ derivative spectral dataset containing the control group (no exposure) and those after 3 years of exposure. In both cases, samples contained the same SiO_2_NPs composition, and were subjected to the cross-validation method using 7 principal components (PCs). The PCA results, which were presented in forms of scores and loading plots, provide an insight into the similarities and differences in chemical composition between the two coating groups both before and after environmental exposure.

### X-ray photoelectron spectroscopy

X-ray photoelectron spectroscopic (XPS) analysis was performed using a Thermo Scientific K-alpha X-ray photoelectron spectrometer (Thermo Fisher Scientific, Victoria, Australia), equipped with a monochromatic X-ray source (Al Ka, h*ν* = 1486.6 eV) operating at 150 W. The spectrometer energy scale was calibrated using the Au 4f7/2 photoelectron peak at a binding energy (BE) of 83.98 eV. During analysis, the samples were flooded with low-energy electrons to counteract any surface charging that may occur. The hydrocarbon component of the C 1s peak (BE = 284.8 eV) was used as a reference for charge correction. Photoelectrons emitted at an angle of 90° to the surface from an area of 700 × 300 μm^2^ were analyzed with 160 eV for survey spectra and then with 20 eV for region spectra. Survey spectra were recorded at intervals of 1.0 eV/step, while the region spectra were taken at intervals of 0.1 eV/step. The Shirley algorithm was used to measure the background core level spectra, and chemically distinct species in the high-resolution regions of the spectra were resolved using synthetic Gaussian–Lorentzian components after the background was removed (using the Thermo Scientific Avantage Data System). High-resolution scans were performed across each of the C 1s peak.

### Raman microspectroscopy

The pure polyester and polyester composites were measured using a Raman micro-spectrometer (WITec, Ulm, Germany) with a laser excitation wavelength of 532 nm (h*ν* = 2.33 eV). A 40× magnification objective (NA = 0.4) was used to characterize the polymer degradation. A grid of 100 × 100 spectra was acquired for a scanning area of 50 × 50 μm^2^. The integration time used for each spectrum was 0.15 s. For each polymer coating sample, Raman mapping measurement was performed in duplicate on two independent samples.

## Results and discussion

### Characterization of surface topography and surface wettability

**Fig 1:**
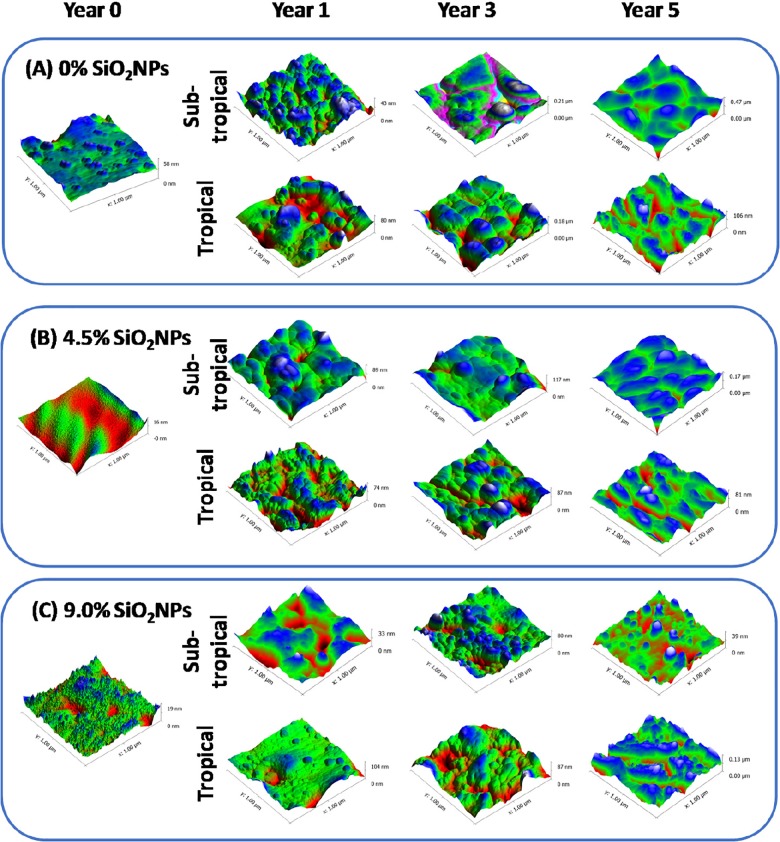
Surface nanotopography of pure polyester and 4.5% and 9% SiO_2_NPs polyester composite coatings on steel substrata under sub-tropical and tropical climate from year 0 to year 5. Atomic force micrographs, over a scanning areas of 1 × 1 μm^2^, showing the change in surface nanotopography of (A) 0% SiO_2_NPs, (B) 4.5% SiO_2_NPs, and (C) 9% SiO_2_NPs after the 5-year exposure to subtropical and tropical climates.

**Fig 2:**
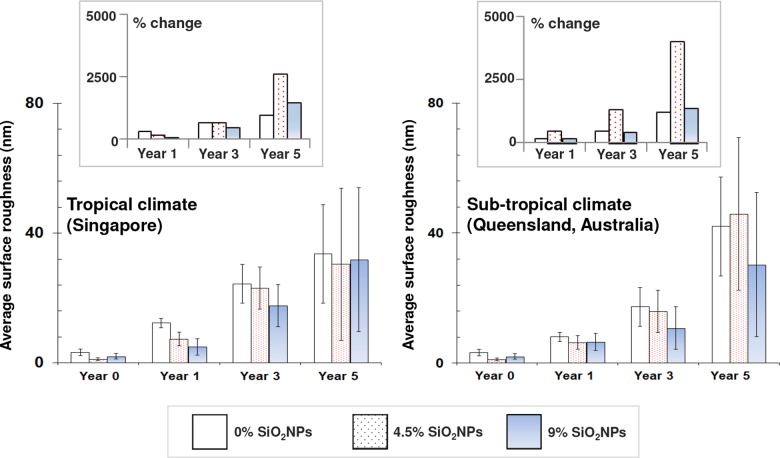
Average nanoscale surface roughness of pure polyester and 4.5% and 9% SiO_2_NPs polyester composite coatings on steel substrata as a function of exposure time from year 0 to year 5, under tropical (*left*) and sub-tropical (*right*) conditions. Data is presented as mean ± standard deviation. *Insets*: change in average roughness values as a function of time.

The surface topography of each substrate coatings was analyzed using AFM over an area of 1 × 1 μm^2^ (Fig 1). The corresponding surface roughness profiles of both pure polyester and polyester composite coatings, containing 4.5% and 9% SiO_2_NPs, are also included in S2 Fig as a function of environmental exposure time (year 0, year 1, year 3 and year 5). As shown in Fig 2, the average roughness of both the pure polyester and polyester composite coatings gradually increased over the 5-year observation period when exposed to the tropical (Singapore) and sub-tropical (Rockhampton, Australia) climate conditions. In particular, the most significant change in the surface roughness was found in 4.5% SiO_2_NPs-embedded polyester composite coatings exposed to the sub-tropical climate (Australia).

**Fig 3:**
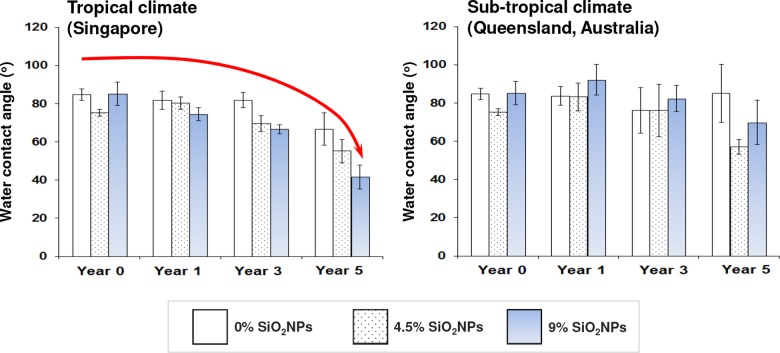
Water contact angle (°) as a function of time for pure polyester and 4.5% and 9% SiO_2_NPs polyester composite coatings on steel surfaces, before and after exposure to tropical (*left*) and sub-tropical (*right*) environments. Data represent mean ± standard deviation.

Contact angle measurement is commonly used for determining the degree of wettability of a surface [11]. Increases in contact angle indicate that changes in surface chemistry take place making the surface become more hydrophobic, whereas a reduction in contact angle signals the surface becoming more hydrophilic. The mean water contact angles for each of the substrate coatings are presented as a function of time in Fig 3. The water contact angles were found to be slightly lower than 90° for all of the substrate coatings prior to exposure, indicating that they were exhibiting marginally hydrophobic behavior, as would be expected for a surface comprised of polyester.

After exposure to the tropical climate (Singapore), both the pure polyester and polyester composite coatings exhibited reducing contact angles as a function of time, indicating that the surface became more hydrophilic. In contrast, under the sub-tropical climate (Australia), the pure polyester coating showed marginal changes in contact angle over the 5-year period. The polymer composite coatings, however, exhibited little if any change in contact angle for three years, after which time a slight reduction in contact angle was observed, indicating that the surface had become more hydrophilic.

### Changes in surface chemistry by environmental exposure observed using synchrotron macro ATR-FTIR and PCA analysis

#### 1. Pure polyester coating

**Fig 4:**
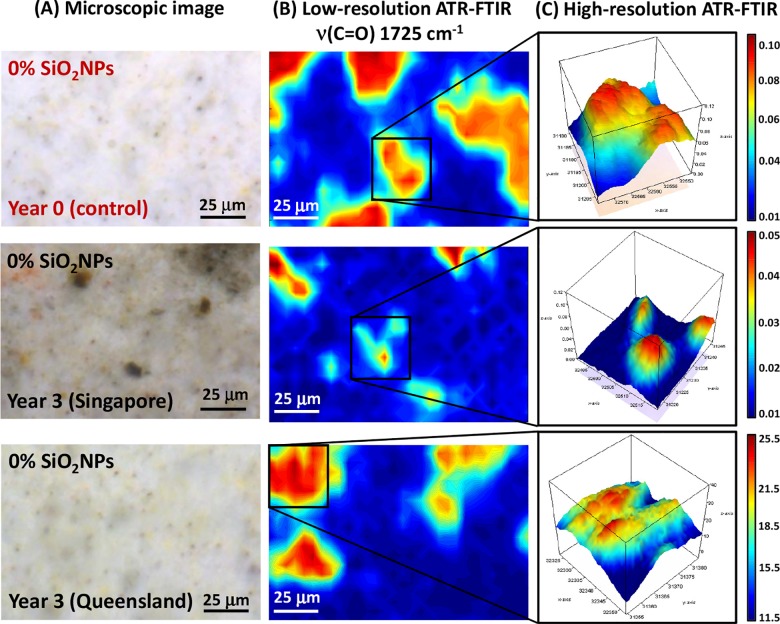
(A) Microscopic images of pure polyester coating surfaces both before and after 3 years of environmental exposure in tropical (Singapore) and sub-tropical (Queensland) climates (from *top* to *bottom*). (B) Low-resolution synchrotron macro ATR-FTIR chemical maps of the *ν*(C = O) band at 1725 cm^-1^, acquired on the same area shown in the corresponding microscopic images. (C) High-resolution synchrotron macro ATR-FTIR 3D chemical maps of the *ν*(C = O) band at 1725 cm^-1^, acquired on specific areas of interest as indicated in (B).

The microscopic images of the pure polyester coating on the steel substrata are presented with the synchrotron macro ATR-FTIR chemical maps, acquired in both low- and high-resolution modes, in Fig 4A. The synchrotron ATR-FTIR chemical maps were produced from integrated area under the band at 1725 cm^-1^, which is attributable to the *ν*(C = O) carbonyl stretching vibration [19], and thereby represent the distribution of the polyester resin used as the second protection layer. In particular, the microscopic images clearly showed distinct differences in the physical appearance of the surface coatings, in terms of their color and surface topography, between the original control group and those exposed to the tropical (Singapore) and sub-tropical (Queensland) climates for 3 years. This physical evidence suggested the formation of a surface chemistry that prompted degradation of the coating material, which appeared to be in an advanced stage for the tropical samples compared to the sub-tropical environment. This difference in surface chemistry is also supported by a substantial decrease in the *ν*(C = O) carbonyl stretching intensity over a much larger surface in the tropical exposure samples compared to those of the sub-tropical climate, as seen via their corresponding low-resolution maps (Fig 4B). Therefore, the observed surface discoloration could be indicative of chemical reaction(s) having taken place that directly affected the carbonyl (C = O) bonds in the polyester coating layer.

**Fig 5:**
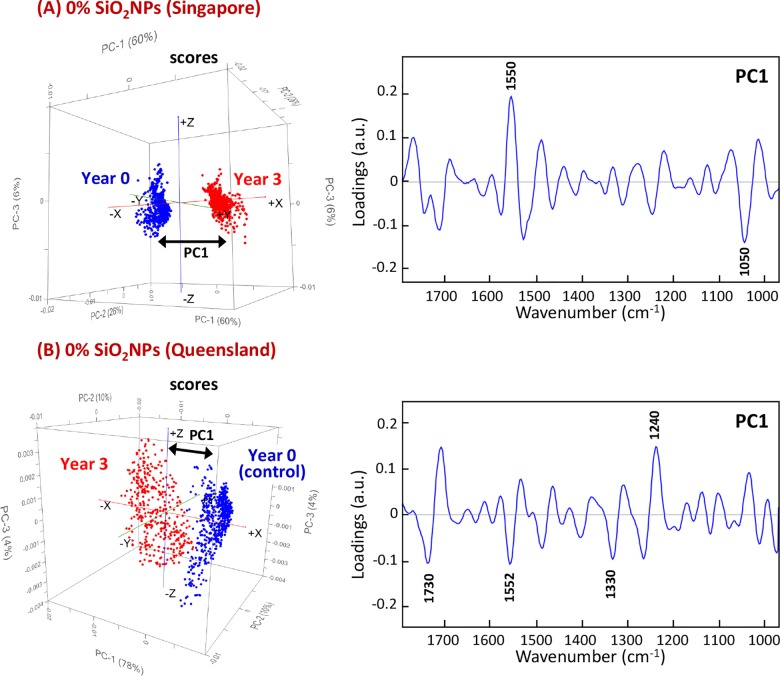
PCA score (*left*) and loading (*right*) plots, based on high-resolution synchrotron macro ATR-FTIR spectra of pure polyester coatings, showing the projection against the first PC (PC1) that explains the majority of the spectral variation between the control sample (year 0) and the samples after 3 years of exposure to (A) tropical Singapore and (B) sub-tropical Queensland climates.

In this study, the PCA results were obtained using the spectral window in the range of 1790–970 cm^-1^, which includes the regions covering the spectral features characteristic of polyester and melamine resins, which are commonly used as cross-linkers [19,20]. Initially, the PCA was performed to allow a differentiation to be made between the spectral patterns of the exposed polyester coatings and their corresponding control group. The resultant score plots shown in Fig 5 clearly revealed the distinct separation of clusters of spectra according to the samples exposed to different climate conditions, along the PC1 axis. Therefore, their corresponding PC1 loadings plots were used to identify the key differences in chemical composition that were responsible for the separation.

By comparing the respective PC1 loading features, it was found that the major changes in molecular structure that were seen to have taken place in the pure polyester coatings exposed to a tropical climate were distinctively different to those exposed to the sub-tropical environment. The most prominent changes in the molecular structure of the coatings exposed to the tropical climate were seen by changes in the vibrational modes at 1550 and 1050 cm^-1^, which were attributed to the *ν*(C = N) stretching mode of triazine ring vibration and the *ν*(C-O-C) stretching modes of the ether groups in the melamine resin, respectively [21]. The PC1 loadings of the samples under sub-tropical exposure, on the other hand, indicated a number of changes in the chemical bonds involving the *ν*(C = O) stretching mode of polyester resin, *ν*(C = N) stretching mode of the triazine ring vibration, δ(C-H) bending mode of methylene groups and δ(O-H), corresponding to deformation in the peaks at 1730, 1552, 1330 and 1240 cm^-1^, respectively [21].

To gain a better understanding of the chemical bonds observed in the polyester coatings as a result of environmental exposure, the climate statistics of Singapore and Queensland were considered. These included temperature, rainfall, % humidity and UV index (S1 Fig). These climate statistics indicated that temperature, % humidity and UV index in Singapore were relatively constant over the entire year, with the exception that the rainfall was substantially greater from November to January. Conversely, these four climate parameters recorded for Queensland (Rockhampton) appeared to significantly fluctuate across the year. The total rainfall in Queensland were only one third of those experienced in Singapore, whereas the average UV index in Queensland was noticeably higher than that recorded in Singapore.

The combination of yearly climate fluctuation, low rainfall and high UV index could be the key factor responsible for changes in the surface chemistry in the polyester coatings exposed to the sub-tropical environment, as evidenced through the loading features presented in Fig 5. Based on the statistical climate data, the constantly high level of rainfall and humidity throughout the year in Singapore potentially played an important role in causing changes in surface chemistry, as seen in variations of the *ν*(C = N) stretching mode of triazine ring vibration and the *ν*(C-O-C) stretching modes of ether groups. These results are consistent with the fact that the triazine ring is known to be very sensitive to hydrolysis by water, and therefore more prone to degradation in high humidity environments [22,23].

#### 2. SiO_2_NPs-embedded polyester composite coating at 4.5% SiO_2_NPs

**Fig 6:**
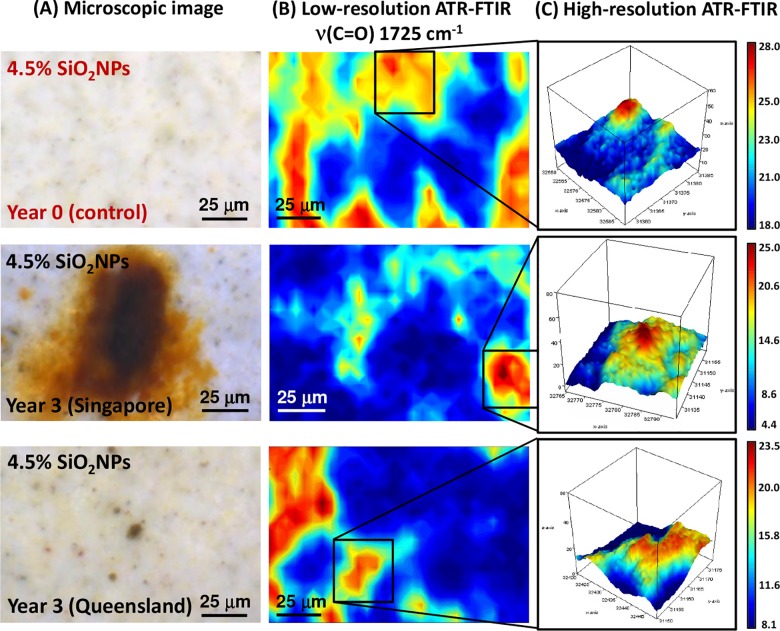
(A) Microscopic images of polyester composite coating surfaces (4.5% SiO_2_NPs) before and after 3 years of environmental exposure to tropical (Singapore) and sub-tropical (Queensland) climates (from *top* to *bottom*). (B) Low-resolution synchrotron macro ATR-FTIR chemical maps of the *ν*(C = O) band at 1725 cm^-1^, acquired on the same area shown in the corresponding microscopic images. (C) High-resolution synchrotron macro ATR-FTIR 3D chemical maps of the *ν*(C = O) band at 1725 cm^-1^, acquired on specific areas of interest as indicated in (B).

Polyester composite coatings containing 4.5% SiO_2_NPs were subjected to the same study as that used for the pure polyester coatings. The microscopic images in Fig 6A highlight substantial changes in the physical appearance of the polyester composite coatings as a result of exposure to the tropical climate in Singapore, compared to the control samples. Clearly visible is large, rust-like discoloration at various locations on the composite surface. Based on the corresponding low-resolution map of the *ν*(C = O) carbonyl stretching vibration in Fig 6B, these deep orange areas possessed very low intensity within the carbonyl band, suggesting an alteration in the carbonyl bonding structure in the polyester coating. In contrast, those exposed to sub-tropical conditions presented only subtle levels of discoloration in the surface.

**Fig 7:**
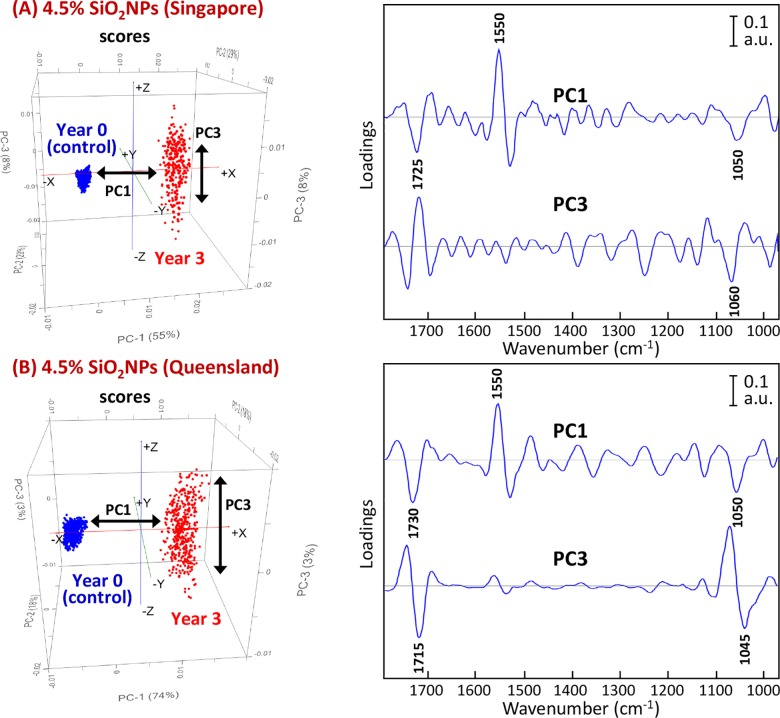
PCA score (*left*) and loading (*right*) plots based on high-resolution synchrotron macro ATR-FTIR spectra of 4.5% SiO_2_NPs polyester composite coatings showing the projection against the first PC (PC1), which explains the majority of the spectral variation between their control and after 3 years of exposure to (A) tropical Singapore and (B) sub-tropical Queensland climates. Note that the PC3 loadings plot is also included for comparison.

For the samples exposed to the tropical environment, the component that was most influential in causing the separation along the PC1 axis was the *ν*(C = N) stretching mode of the triazine ring vibration, as indicated by the PC1 loadings at 1550 cm^-1^ (Fig 7). Moderate contributions also arose from the *ν*(C = O) carbonyl stretches of the polyester resin and the *ν*(C-O-C) stretching modes of ether groups at 1725 and 1050 cm^-1^, respectively. Alteration of these specific bonding structures could be the key factor that led to the presence of the rust-like stains. In addition, the score plot presented in Fig 7A revealed that a cluster of the polyester composite coatings at 4.5% SiO_2_NPs after 3 years of exposure to the tropical environment resulted in a longitudinal dispersion along the PC3 axis, which was not observed for the pure polyester coatings. According to the corresponding PC3 loadings, dispersion of the data cluster was caused by a large variation in the *ν*(C = O) carbonyl stretches in the polyester resin and the *ν*(C-O-C) stretches of the ether group.

For the polyester composite coatings subjected to sub-tropical exposure, a similar loading pattern was observed. The *ν*(C = N) stretching mode of the triazine ring vibration in the melamine resin at 1550 cm^-1^ was the strongest contributor to the separation, followed by the *ν*(C = O) carbonyl stretches of the polyester resin and the *ν*(C-O-C) stretches of the ether group, at 1730 and 1050 cm^-1^, respectively. The longitudinal dispersion observed along the PC3 axis within the exposed polyester composite samples also suggested the presence of a large variation in the *ν*(C = O) carbonyl bonds in the polyester resin and the *ν*(C-O-C) stretches of the ether group, each leading to the formation of dark spots and yellow stains seen in the microscopic images.

#### 3. SiO_2_NPs-embedded polyester composite coating at 9% SiO_2_NPs

The microscopic images and PCA results obtained for the 9% SiO_2_NPs polyester composite coatings after 3 years of exposure to tropical and sub-tropical environments appeared to be closely similar to those observed for the of the 4.5% SiO_2_NPs polyester composite coatings.

**Fig 8:**
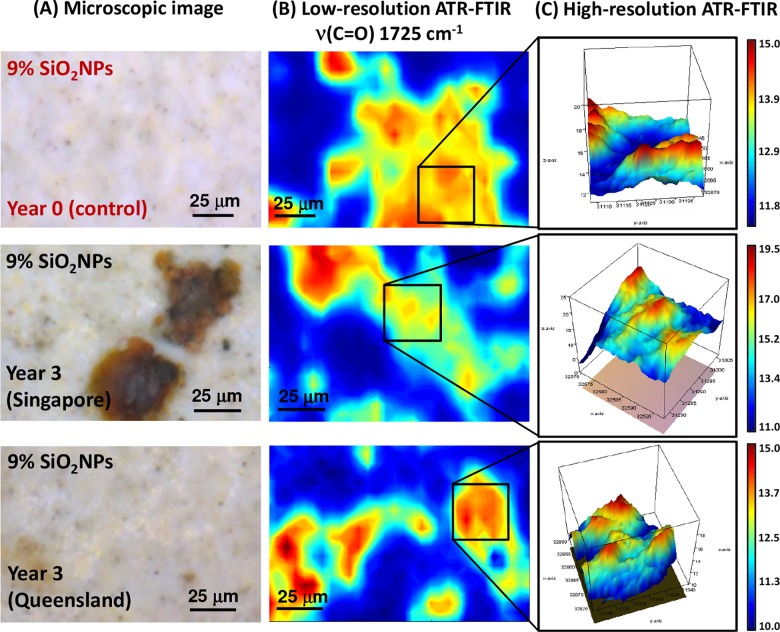
(A) Microscopic images of 9% SiO_2_NPs polyester composite coating surfaces before and after 3 years of environmental exposure to tropical (Singapore) and sub-tropical (Queensland) climates (from *top* to *bottom*). (B) Low-resolution synchrotron macro ATR-FTIR chemical maps of the *ν*(C = O) band at 1725 cm^-1^, acquired on the same area shown in the corresponding microscopic images. (C) High-resolution synchrotron macro ATR-FTIR 3D chemical maps of the *ν*(C = O) band at 1725 cm^-1^, acquired on specific areas of interest as indicated in (B).

As illustrated in Fig 8A, the polyester composite coating samples exposed to the tropical climate revealed the formation of large, rust-like discolorations at various locations of the composite surface. These stained areas corresponded to changes in the low carbonyl (C = O) band intensities in the low-resolution map, as shown in Fig 8B. This suggested that a similar chemical alteration of the carbonyl bonding structure occurred when SiO_2_NPs was incorporated into the polyester/melamine layer, which appeared to be independent of the SiO_2_NPs composition.

**Fig 9:**
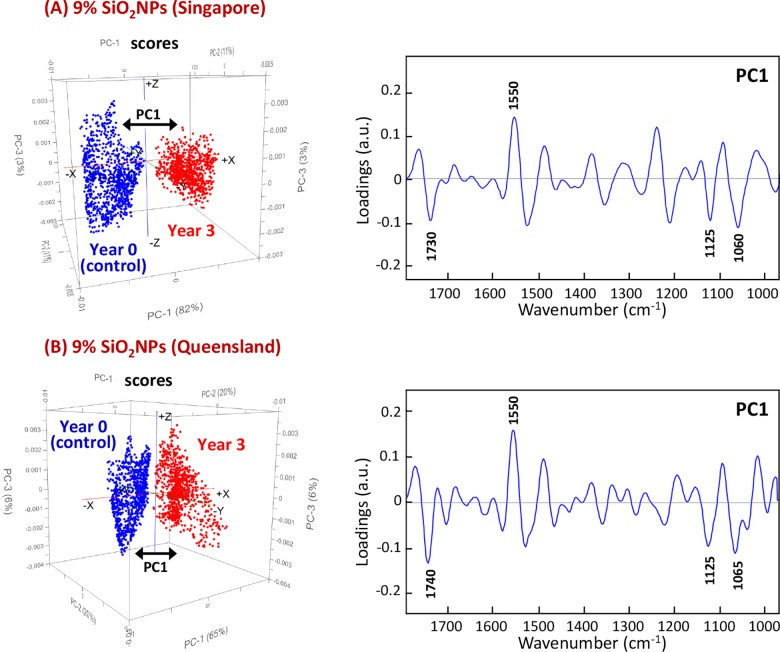
PCA score (*left*) and loading (*right*) plots based on high-resolution synchrotron macro ATR-FTIR spectra of 9% SiO_2_NPs polyester composite coatings showing the projection against the first PC (PC1), which explains the majority of the spectral variation before and after 3 years of exposure to (A) tropical Singapore and (B) sub-tropical Queensland climates.

The PCA scores and loading plots of the 9% SiO_2_NPs polyester composite coatings are presented in Fig 9. The results for these composite coatings are very similar for the samples exposed to tropical and sub-tropical climates. In both cases, the chemical groups that had been altered by the environment were clearly separated from their control counterparts. Once again, changes in the groups corresponding to the *ν*(C = N) stretching mode of the triazine ring vibration in the melamine resin and the *ν*(C = O) carbonyl stretching modes in the polyester resin at 1550 and 1730 cm^-1^ were the most profound differentiators between the groups. There was also a moderate impact in the loadings at 1065 and 1125 cm^-1^, which corresponded to the stretching vibration of *ν*(C-O-C) of ethers and *ν*(C-O) of aliphatic groups, respectively [21].

### Climate-induced effect on functional groups observed using XPS and raman techniques

In recent years, XPS technique has been demonstrated to be a useful analytical tool for investigating crosslinking and segregation mechanisms in hexamethoxymethyl melamine/polyester resins, as well as surface modification of a polyester non-woven with a dielectric barrier discharge in air at medium pressure [24,25]. Accordingly, the XPS technique was employed in this study to further investigate the effect of the environmental exposure on functional groups of pure polyester and polyester composite coatings. Deconvolution analysis approach was performed on the acquired C 1s high resolution spectra (S3 Fig). As shown. the deconvoluted spectra of both pure polyester and polyester composites revealed three peaks at 284.6, 286.1 and 289.0 eV, corresponding to C-C/C-H, C-O/C-OH, and C = O/O-C = O functional groups, respectively. By a comparison, the exposure to the high UV/high humidity climates resulted in substantial decreases in C-C/C-H and C-O/C-OH groups, while the C = O/O-C = O functional groups remained to be relatively the same in all cases.

Subsequently, Raman mapping microspectroscopic measurement was performed on the same polymer/polymer composite samples, to acquire additional molecular information complimentary to synchrotron FTIR data. The resultant Raman images of the pure polyester (0% SiO_2_NPs) and the other two polyester composites (4.5% and 9% SiO_2_NPs) are illustrated in Supporting Information S4–S6 Figs, respectively, based on integrated intensities in the range of 100–700 cm^-1^. The two strong Raman peaks at 450 and 625 cm^-1^ are characteristic of ester groups in the polymer network structure. In particular, Raman images obtained from the samples after 3 years of exposure to both climate conditions, indicate black pits on the surfaces. The corresponding Raman spectra extracted from the areas of the black pits showed the disappearance of the two characteristic ester bands at 450 and 625 cm^-1^, providing an additional supporting evidence of polymer degradation on the surfaces of these coating layers as a result of prolonged exposure to high UV/humidity climates.

## Conclusions

In this work, spatially resolved synchrotron macro ATR-FTIR microspectroscopy, in combination with a multivariate data analysis approach, was demonstrated to be a powerful technique in the characterization of surface coating materials at the molecular level. The technique provided not only a better understanding of changes taking place in the molecular distribution over time, through high-resolution chemical mapping technology, but it also provided valuable insights via PCA into the molecular changes taking place.

The results obtained using this combined technique provided evidence of changes occurred as a result of environmental exposure, in the molecular structure of the polymer. Exposure of the pure polyester coating to a tropical climate resulted in different changes in molecular structure than those samples exposed to a sub-tropical environment. Whilst changes in the triazine ring and ether group in the melamine resin were two prominent changes in the samples exposed to a tropical climate, the samples exposed to a sub-tropical environment underwent changes in the carbonyl group of the polyester resin in addition to changes in the hydroxyl groups, the triazine ring and hydrocarbon groups.

Incorporating SiO_2_NPs into the polyester coating resulted in similar changes in the surface chemistry of the surface coatings exposed to both tropical and sub-tropical environments after 3 years of exposure. The *ν*(C = N) stretching mode of triazine ring vibration in the melamine resins was found to be the most influential component that caused the separation between the control and the exposed samples, which is likely to be due to the triazine ring being hydrolyzed in humid environments. At the higher loading of SiO_2_NPs at 9%, a large number of chemical bonds associated to melamine/polyester networking structure were shown to be affected, resulting in substantial surface degradation being observed after long-term exposure to a high UV/high humidity environment.

## Supporting information

S1 Fig Monthly climate data of tropical (Singapore) and sub-tropical (Rockhampton, Queensland, Australia) regions. Data were obtained from Meteorological Service Singapore (http://www.weather.gov.sg) and Bureau of Meteorology, Australian Government (http://www.bom.gov.au).
(PDF)

S2 Fig Surface roughness profile of pure polyester (0% SiO_2_NPs) and polyester composite coatings (4.5% and 9% SiO_2_NPs) on steel substrata under sub-tropical and tropical climates from year 0 to year 5. Their corresponding surface profiles observed using AFM technique (Fig 1) showing the change in surface nanotopography of (A) 0% SiO_2_NPs, (B) 4.5% SiO_2_NPs, and (C) 9% SiO_2_NPs after the 5-year exposure to sub-tropical and tropical climates.
(PDF)

S3 Fig Deconvolution analyses of XPS C 1s high resolution spectra of pure polyester (0% SiO_2_NPs) and polyester composite coatings (4.5% and 9% SiO_2_NPs) on steel substrata under sub-tropical and tropical climates after 3 years of environmental exposure. (PDF)

S4 Fig Representative Raman microspectroscopic spectra (*left*) and their corresponding Raman images based on integrated intensities in the range of 100–700 cm^-1^ (*right*), observed on pure polyester coating surfaces (0% SiO_2_NPs) before and after 3 years of environmental exposure in sub-tropical and tropical climates (from *top* to *bottom*). Note that the representative Raman spectra were extracted from the locations indicated by arrows on the corresponding images.
(PDF)

S5 Fig Representative Raman microspectroscopic spectra (*left*) and their corresponding Raman images based on integrated intensities in the range of 100–700 cm^-1^ (*right*), observed for 4.5% SiO_2_NPs-embedded polyester composite coatings before and after 3 years of environmental exposure in sub-tropical and tropical climates (from *top* to *bottom*). Note that the representative Raman spectra were extracted from the locations indicated by arrows on the corresponding images.
(PDF)

S6 Fig Representative Raman microspectroscopic spectra (*left*) and their corresponding Raman images based on integrated intensities in the range of 100–700 cm^-1^ (*right*), observed for 9% SiO_2_NPs-embedded polyester composite coatings before and after 3 years of environmental exposure in sub-tropical and tropical climates (from *top* to *bottom*). Note that the representative Raman spectra were extracted from the locations indicated by arrows on the corresponding images.
(PDF)

## References

[1] Li LJ, Pi PH, Wang LS, Wen XF, Cheng J, Yang ZR A review of surface modification of aluminum pigments applied in coatings. Corrosion and Protection. 2009; 30: 519–522. https://doi.org/10.1016/j.cis.2006.11.010

[2] Kim S, Moon GH, Kim G, Kang U, Park H, Choi W TiO_2_ complexed with dopamine-derived polymers and the visible light photocatalytic activities for water pollutants. Journal of Catalysis. 2017; 346: 92–100. https://doi.org/10.1016/j.jcat.2016.11.027

[3] Moreira DC, Braga NR, Sphaier LA, Nunes LCS Size effect on the thermal intensification of alumina-filled nanocomposites. Journal of Composite Materials. 2016; 50: 3699–3707. https://doi.org/10.1177/0021998315624253

[4] Nikolic M, Sanadi AR, Löf D, Barsberg ST Influence of colloidal nano-silica on alkyd autoxidation. Journal of Materials Science. 2017; 52: 7158–7165. https://doi.org/10.1007/s10853-017-0951-7

[5] Hollamby MJ, Fix D, Donch I, Borisova D, Mohwald H, Shchukin D Hybrid polyester coating incorporating functionalized mesoporous carriers for the holistic protection of steel surfaces. Advanced materials. 2011; 23: 1361–1365. https://doi.org/10.1002/adma.201003035 doi: 10.1002/adma.201003035 21400596 10.1002/adma.201003035

[6] Carr GL Resolution limits for infrared microspectroscopy explored with synchrotron radiation. Review of Scientific Instruments. 2001; 72: 1613–1619. http://doi.org/10.1063/1.1347965

[7] Miller LM, Smith RJ Synchrotrons versus globars, point-detectors versus focal plane arrays: Selecting the best source and detector for specific infrared microspectroscopy and imaging applications. Vibrational Spectroscopy. 2005; 38: 237–240. http://doi.org/10.1016/j.vibspec.2005.03.010

[8] Tobin MJ, Bambery KR, Martin DE, Puskar L, Beattie DA, Ivanova EP, et al Attenuated Total Reflection FTIR Microspectroscopy at the Australian Synchrotron. OSA Technical Digest (online); 2016 2016/11/14; Leipzig Optical Society of America. pp. FTu2E.5.

[9] Truong VK, Stefanovic M, Maclaughlin S, Tobin M, Vongsvivut J, Al Kobaisi M, et al The evolution of silica nanoparticle-polyester coatings on surfaces exposed to sunlight. Journal of Visualized Experiments: JoVE. 2016: e54309 https://doi.org/10.3791/54309 10.3791/54309 PMC5092198 27768041

[10] Crawford RJ, Webb HK, Truong VK, Hasan J, Ivanova EP Surface topographical factors influencing bacterial attachment. Advances in Colloid and Interface Science. 2012; 179–182: 142–149. http://doi.org/10.1016/j.cis.2012.06.015 doi: 10.1016/j.cis.2012.06.015 22841530 10.1016/j.cis.2012.06.015

[11] Truong VK, Lapovok R, Estrin YS, Rundell S, Wang JY, Fluke CJ, et al The influence of nano-scale surface roughness on bacterial adhesion to ultrafine-grained titanium. Biomaterials. 2010; 31: 3674–3683. http://doi.org/10.1016/j.biomaterials.2010.01.071 doi: 10.1016/j.biomaterials.2010.01.071 20163851 10.1016/j.biomaterials.2010.01.071

[12] Savitzky A, Golay MJE Smoothing and differentiation of data by simplified least squares procedures. Analytical Chemistry. 1964; 36: 1627–1639. https://doi.org/10.1021/ac60214a047

[13] Rieppo L, Saarakkala S, Narhi T, Helminen HJ, Jurvelin JS, Rieppo J Application of second derivative spectroscopy for increasing molecular specificity of Fourier transform infrared spectroscopic imaging of articular cartilage. Osteoarthritis Cartilage. 2012; 20: 451–459. https://doi.org/10.1016/j.joca.2012.01.010 doi: 10.1016/j.joca.2012.01.010 22321720 10.1016/j.joca.2012.01.010

[14] Vongsvivut J, Heraud P, Gupta A, Puri M, McNaughton D, Barrow CJ FTIR microspectroscopy for rapid screening and monitoring of polyunsaturated fatty acid production in commercially valuable marine yeasts and protists. Analyst. 2013; 138: 6016–6031. https://doi.org/10.1039/C3AN00485F doi: 10.1039/c3an00485f 23957051 10.1039/c3an00485f

[15] Naumann D, Labischinski H, Giesbrecht P (1990) The characterization of microorganisms by Fourier transform infrared spectroscopy (FT-IR) In: Nelson WH, editor. Modern Techniques for Rapid Microbiological Analysis. Weinheim: VCH Verlag Chemie.

[16] Kohler A, Afseth NK, Martens H (2010) Chemometrics in Biospectroscopy In: Li-Chan E, Griffiths PR, Chalmers JM, editors. Applications of Vibrational Spectroscopy in Food Science. Chichester, UK: John Wiley & Sons pp. 89–108.

[17] Kohler A, Kirschner C, Oust A, Martens H Extended multiplicative signal correction as a tool for separation and characterization of physical and chemical information in Fourier transform infrared microscopy images of cryo-sections of beef loin. Applied Spectroscopy. 2005; 59: 707–716. https://doi.org/10.1366/0003702054280649 doi: 10.1366/0003702054280649 16053536 10.1366/0003702054280649

[18] Kohler A, Sule-Suso J, Sockalingum GD, Tobin M, Bahrami F, Yang Y, et al Estimating and correcting Mie scattering in synchrotron-based microscopic Fourier transform infrared spectra by extended multiplicative signal correction. Applied Spectroscopy. 2008; 62: 259–266. https://doi.org/10.1366/000370208783759669 doi: 10.1366/000370208783759669 18339231 10.1366/000370208783759669

[19] Hirayama T, Urban MW Distribution of melamine in melamine/polyester coatings; FT-IR spectroscopic studies. Progress in Organic Coatings. 1992; 20: 81–96. http://doi.org/10.1016/0033-0655(92)85006-H

[20] Gamage NJW, Hill DJT, Lukey CA, Pomery PJ Distribution of melamine in polyester–melamine surface coatings cured under nonisothermal conditions. Journal of Polymer Science Part A: Polymer Chemistry. 2004; 42: 83–91. https://doi.org/10.1002/pola.11006

[21] Merline DJ, Vukusic S, Abdala AA Melamine formaldehyde: Curing studies and reaction mechanism. Polymer Journal. 2013; 45: 413–419. https://doi.org/10.1038/pj.2012.162

[22] Smolin EM, Rapoport L (1959) *s*-Triazines and Derivatives In: Weissberger A, editor. The Chemistry of Heterocyclic Compounds. New York: InterScience Publishers, Inc. pp. 644.

[23] Neunhoeffer H, Wiley PF (1978) Polymers Containing the 1,2,4-Triazine Nucleus In: Neunhoeffer H, Wiley PF, editors. Chemistry of Heterocyclic Compounds: Chemistry of 1,2,3-Triazines and 1,2,4-Triazines, Tetrazines, and Pentazin. New York: John Wiley & Sons, Inc. pp. 1005–1072.

[24] Perruchot C, Abel ML, Watts JF, Lowe C, Maxted JT, White RG High-resolution XPS study of crosslinking and segregation phenomena in hexamethoxymethyl melamine-polyester resins. Surface and Interface Analysis. 2002; 34: 570–574. https://doi.org/10.1007/10.1002/sia.1362

[25] De Geyter N, Morent R, Leys C Surface modification of a polyester non-woven with a dielectric barrier discharge in air at medium pressure. Surface and Coatings Technology. 2006; 201: 2460–2466. https://doi.org/10.1016/j.surfcoat.2006.04.004

