# Supplementary material for: Synchrotron macro ATR-FTIR microspectroscopic analysis of silica nanoparticle-embedded polyester coated steel surfaces subjected to prolonged UV and humidity exposure
Source: PLoS One. 2017 Dec 18;12(12):e0188345. doi: 10.1371/journal.pone.0188345 (PMC5734741; doi:10.1371/journal.pone.0188345)
Supplement: S1 Fig — Data were obtained from Meteorological Service Singapore (http://www.weather.gov.sg) and Bureau of Meteorology, Australian Government (http://www.bom.gov.au). (PDF) [file pone.0188345.s001.pdf]

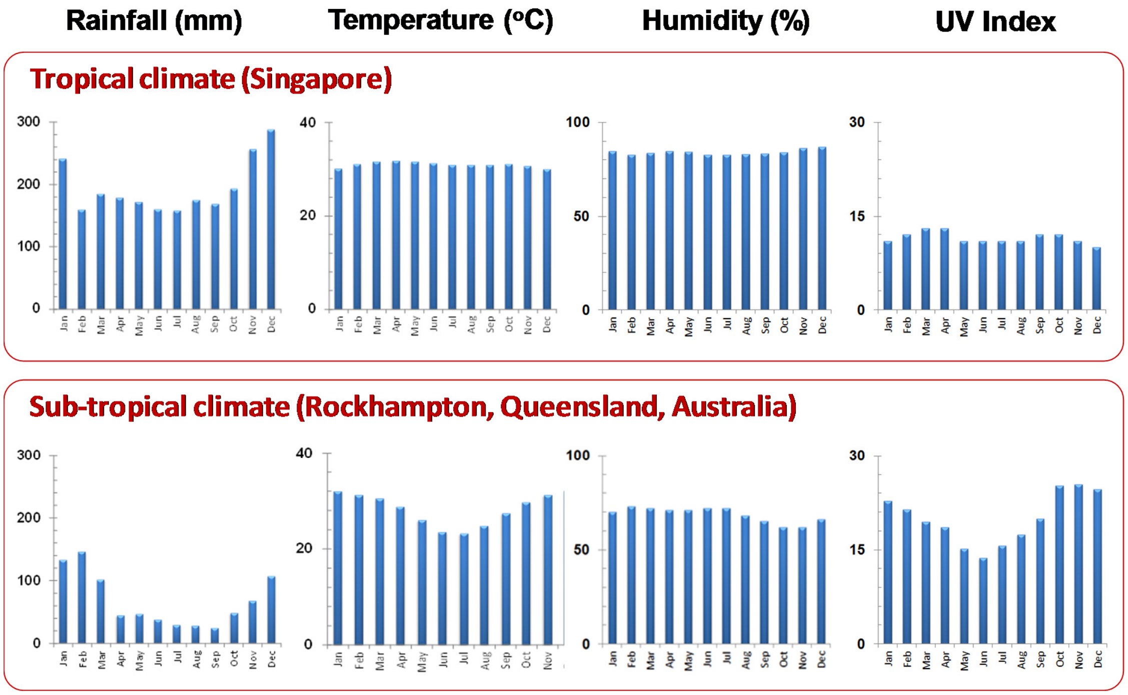

S1 Fig. Monthly climate data of tropical (Singapore) and sub-tropical (Rockhampton, Queensland, Australia) regions. Data were obtained from Meteorological Service Singapore (http://www.weather.gov.sg) and Bureau of Meteorology, Australian Government (http://www.bom.gov.au).
