# Supplementary material for: Synchrotron macro ATR-FTIR microspectroscopic analysis of silica nanoparticle-embedded polyester coated steel surfaces subjected to prolonged UV and humidity exposure
Source: PLoS One. 2017 Dec 18;12(12):e0188345. doi: 10.1371/journal.pone.0188345 (PMC5734741; doi:10.1371/journal.pone.0188345)
Supplement: S2 Fig — Their corresponding surface profiles observed using AFM technique (Fig 1) showing the change in surface nanotopography of (A) 0% SiO2NPs, (B) 4.5% SiO2NPs, and (C) 9% SiO2NPs after the 5-year exposure to sub-tropical and tropical climates. (PDF) [file pone.0188345.s002.pdf]

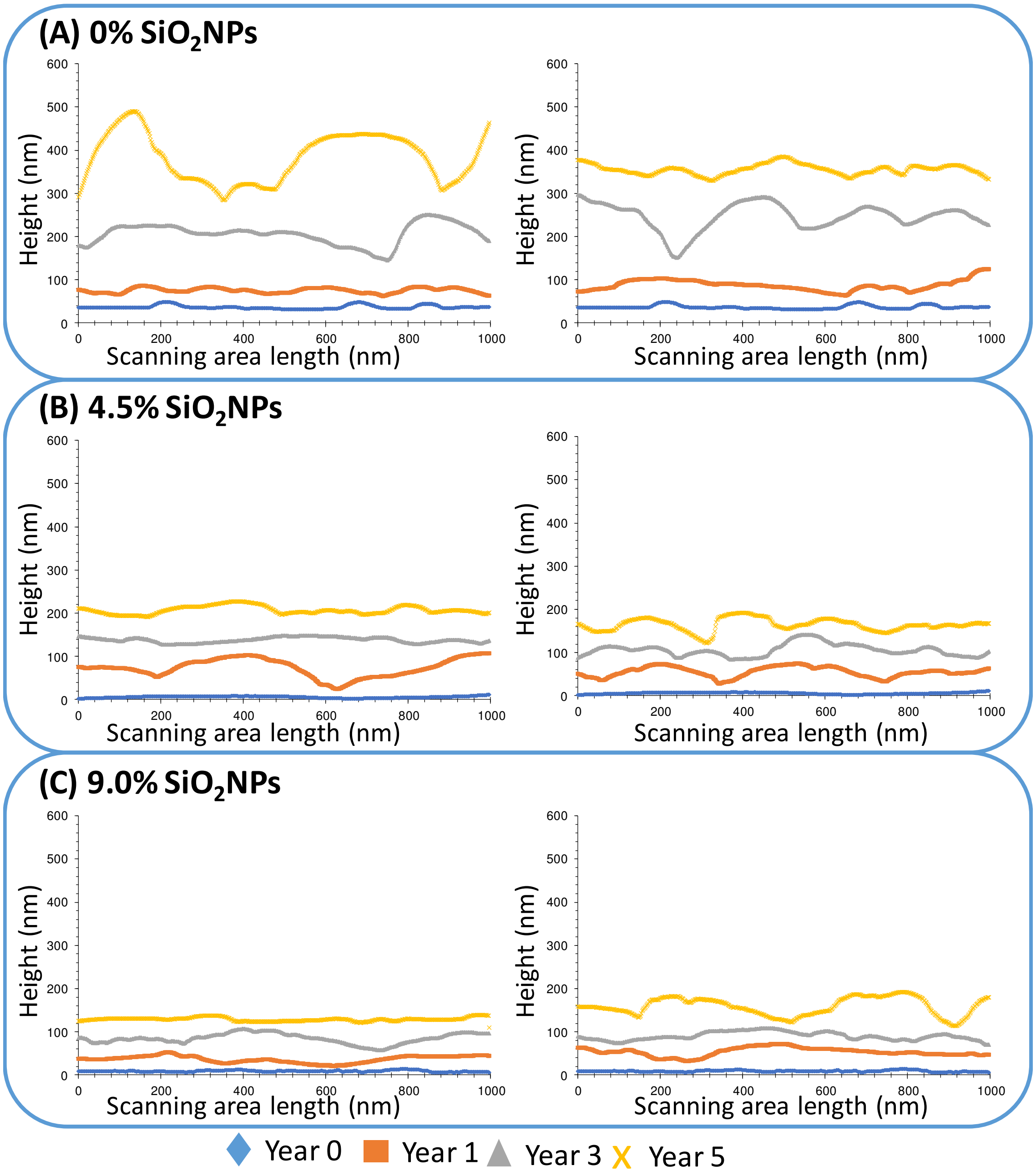

S2 Fig. Surface roughness profile of pure polyester (0% SiO_2NPs) and polyester composite coatings (4.5% and 9% SiO_2NPs) on steel substrata under subtropical and tropical climate from year 0 to year 5. Corresponding surface profiles of atomic force micrographs (Fig. 1) showing the change in surface nanotopography of (A) 0% SiO_2NPs, (B) 4.5% SiO_2NPs, and 9% SiO_2NPs after the 5-year exposure to sub-tropical and tropical climates.
