# Supplementary material for: Synchrotron macro ATR-FTIR microspectroscopic analysis of silica nanoparticle-embedded polyester coated steel surfaces subjected to prolonged UV and humidity exposure
Source: PLoS One. 2017 Dec 18;12(12):e0188345. doi: 10.1371/journal.pone.0188345 (PMC5734741; doi:10.1371/journal.pone.0188345)
Supplement: S3 Fig — (PDF) [file pone.0188345.s003.pdf]

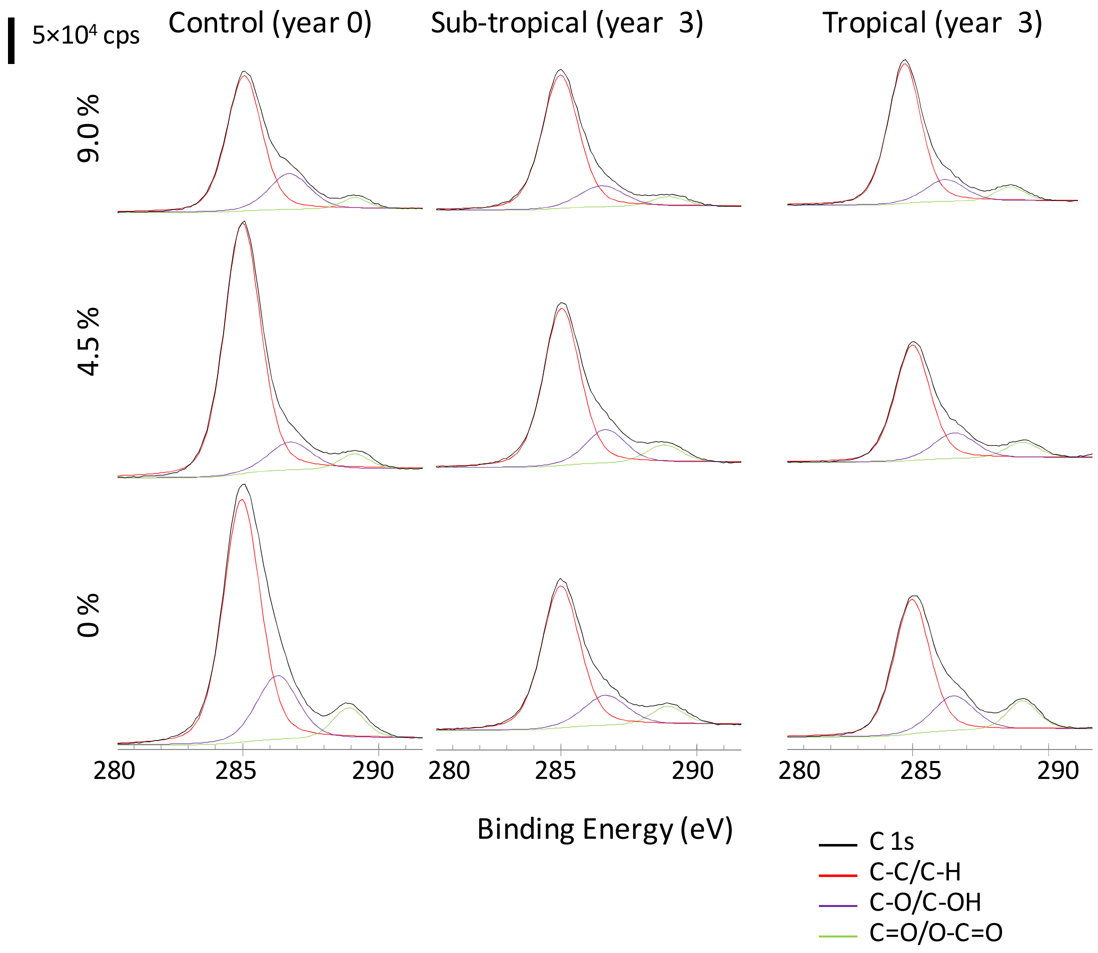

S3 Fig. Deconvolution analyses of XPS C 1s high resolution spectra of pure polyester (0% SiO_2NPs) and polyester composite coatings (4.5% and 9% SiO_2NPs) on steel substrata under sub-tropical and tropical climates after 3 years of environmental exposure.
