# Supplementary material for: Synchrotron macro ATR-FTIR microspectroscopic analysis of silica nanoparticle-embedded polyester coated steel surfaces subjected to prolonged UV and humidity exposure
Source: PLoS One. 2017 Dec 18;12(12):e0188345. doi: 10.1371/journal.pone.0188345 (PMC5734741; doi:10.1371/journal.pone.0188345)
Supplement: S5 Fig — Note that the representative Raman spectra were extracted from the locations indicated by arrows on the corresponding images. (PDF) [file pone.0188345.s005.pdf]

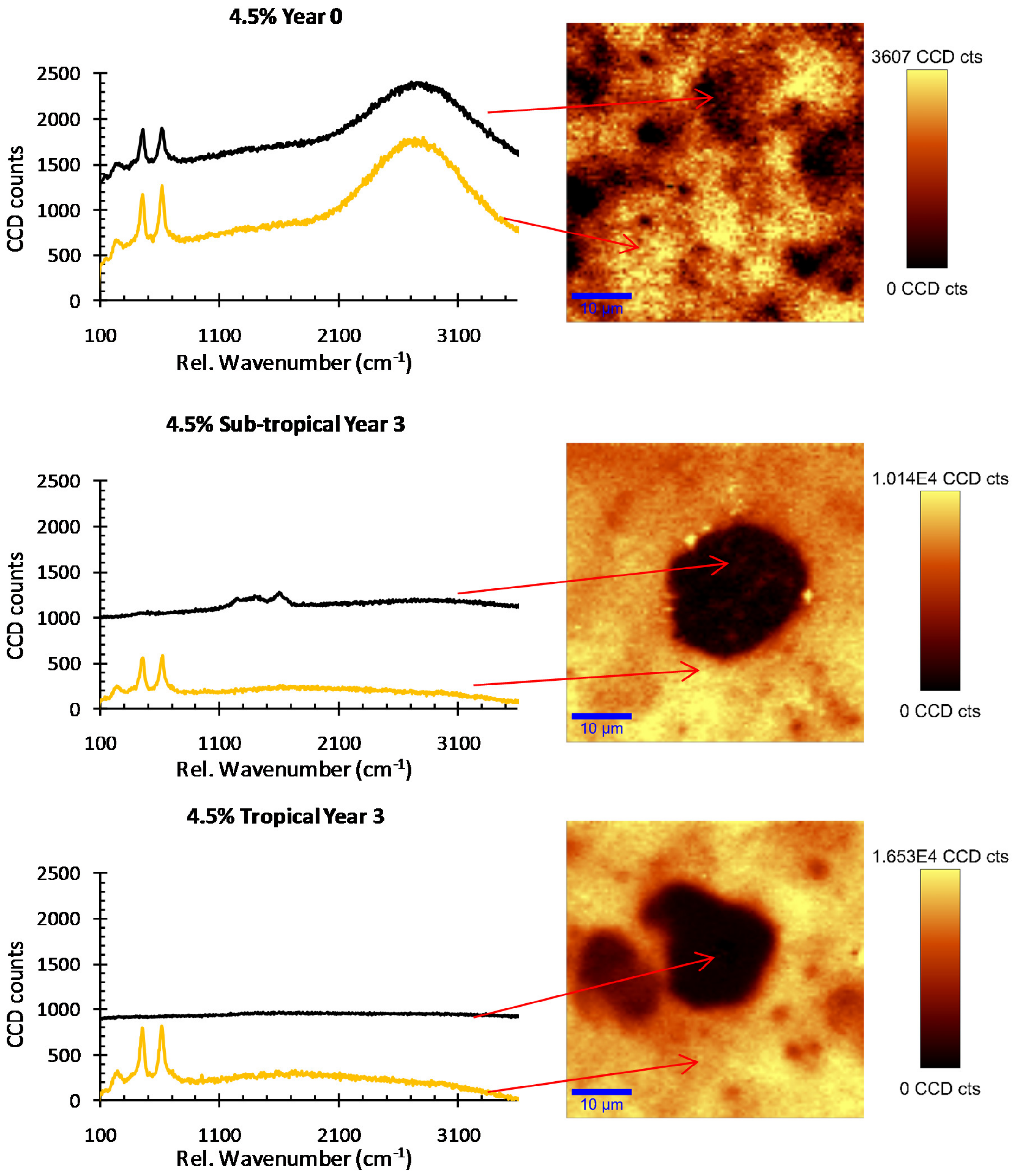

S5 Fig. Representative Raman microspectroscopic spectra (*left*) and their corresponding Raman images based on integrated intensities in the range of 100-700 cm^{-1} (*right*), observed for 4.5% SiO_2NPs-embedded polyester composite coatings before and after 3 years of environmental exposure in sub-tropical and tropical climates (from *top* to *bottom*). Note that the representative Raman spectra were extracted from the locations indicated by arrows on the corresponding images.
